# Urban Tree Species Classification Using a WorldView-2/3 and LiDAR Data Fusion Approach and Deep Learning

**DOI:** 10.3390/s19061284

**Published:** 2019-03-14

**Authors:** Sean Hartling, Vasit Sagan, Paheding Sidike, Maitiniyazi Maimaitijiang, Joshua Carron

**Affiliations:** Department of Earth and Atmospheric Sciences, Saint Louis University, St. Louis, MO 63108, USA; sean.hartling@slu.edu (S.H.); sidike.paheding@slu.edu (P.S.); mason.maimaitijiang@slu.edu (M.M.); Joshua.Carron@slu.edu (J.C.)

**Keywords:** deep learning, dense convolutional network (DenseNet), convolutional neural network (CNN), support vector machine (SVM), random forest (RF), tree species classification, data fusion

## Abstract

Urban areas feature complex and heterogeneous land covers which create challenging issues for tree species classification. The increased availability of high spatial resolution multispectral satellite imagery and LiDAR datasets combined with the recent evolution of deep learning within remote sensing for object detection and scene classification, provide promising opportunities to map individual tree species with greater accuracy and resolution. However, there are knowledge gaps that are related to the contribution of Worldview-3 SWIR bands, very high resolution PAN band and LiDAR data in detailed tree species mapping. Additionally, contemporary deep learning methods are hampered by lack of training samples and difficulties of preparing training data. The objective of this study was to examine the potential of a novel deep learning method, Dense Convolutional Network (DenseNet), to identify dominant individual tree species in a complex urban environment within a fused image of WorldView-2 VNIR, Worldview-3 SWIR and LiDAR datasets. DenseNet results were compared against two popular machine classifiers in remote sensing image analysis, Random Forest (RF) and Support Vector Machine (SVM). Our results demonstrated that: (1) utilizing a data fusion approach beginning with VNIR and adding SWIR, LiDAR, and panchromatic (PAN) bands increased the overall accuracy of the DenseNet classifier from 75.9% to 76.8%, 81.1% and 82.6%, respectively. (2) DenseNet significantly outperformed RF and SVM for the classification of eight dominant tree species with an overall accuracy of 82.6%, compared to 51.8% and 52% for SVM and RF classifiers, respectively. (3) DenseNet maintained superior performance over RF and SVM classifiers under restricted training sample quantities which is a major limiting factor for deep learning techniques. Overall, the study reveals that DenseNet is more effective for urban tree species classification as it outperforms the popular RF and SVM techniques when working with highly complex image scenes regardless of training sample size.

## 1. Introduction

Vegetation has aesthetic, environmental, human health, and economic benefits in urban ecosystems. Trees play an integral role within the urban environment as oxygen producers, improving air quality, mitigating urban heat island effect, and raising property values [1]. Tree species diversity is a vital parameter to characterize urban ecosystems. It is also becoming more and more important for sustainable urban planning. Therefore, spatially-explicit detailed tree species mapping is critical for understanding the value to ecological services, in addition to establishing policies for sustainable urban development [2]. Traditional inventories of urban tree species derived from field surveys and manual interpretation of aerial photographs are costly, time-consuming, and lack the ability to cover large areas [3]. Conversely, remote sensing methods, such as aerial and satellite imagery, can provide timely and detailed data at finer temporal and spatial scales at a lower cost than extensive field sampling. Initial attempts at tree species mapping were limited to broad vegetation coverage or single tree species over homogenous forest stands using moderate spectral and spatial resolution sensors (e.g., Landsat, MODIS, etc.). However, mapping individual tree species in a complex urban environment is challenging due to the fine scale of spatial variation and highly heterogeneous background.

Recently, enhanced remote sensing techniques and high spatial resolution satellite imagery (e.g., IKONOS, WorldView, GeoEye, Planet Labs) have expanded the ability to classify tree species in a complex urban environment [4,5]. Contrary to moderate spatial resolution satellite imagery, individual tree crowns can be distinguished in high spatial resolution imagery [6]. While some of these satellite sensors (e.g., IKONOS, GeoEye) are capable of acquiring <3 m multispectral spatial resolution, they do not have the spectral range and spatial resolution necessary to discriminate the subtle difference in structural and chemical composition between tree species [7]. Compared to the traditional four-spectral band IKONOS and GeoEye, the WorldView-2 (WV2) satellite (DigitalGlobe Inc., Westminster, CO, USA) launched in 2009 has eight spectral bands and a spatial resolution of 0.5 m in the panchromatic band and 2.0 m in the VNIR bands. The four additional bands (coastal, yellow, red-edge and near-infrared 2 bands) increase the ability to adequately distinguish tree species [8,9,10]. The WorldView-3 (WV3) satellite was launched in August 2014 with a 16-band mode that consists of eight VNIR bands, similar to WV2, in addition to eight short-wave infrared (SWIR) bands that may enhance vegetation analysis. However, to our knowledge, no studies have demonstrated the benefits of SWIR bands in detailed mapping of urban tree species.

Airborne Light Detection and Ranging (LiDAR) systems provide highly accurate 3-dimensional (3D) information capable of measuring height, structural characteristics and other biophysical properties of vegetation. Individual or stand-level tree parameters, such as tree height, canopy density, canopy volume, crown shape/width, diameter at breast height (DBH) and Leaf Area Index, can be estimated through the combination of field data with 3D structural information ascertained from intensity/range of individual pulse returns recorded by LiDAR sensors [11]. The structural information derived from LiDAR data can add another contributing dimension to remotely-sensed individual tree analysis, when combined with the biochemical and biophysical information extracted from spectral sensors [3].

The effectiveness of any image classification depends on a variety of considerations, in conjunction with the selection of an appropriate classifier [12]. Parametric classifiers like maximum likelihood classifier (MLC) are not suitable for urban tree species classification because of the highly complex image scenes and limitations when handling high dimensional, multi-source data [13]. Over the past two decades, machine learning algorithms have been developed as a more accurate and efficient alternative to conventional parametric classifiers, when dealing with highly dimensional and complex image data. Popular non-parametric classifiers, such as Support Vector Machine (SVM) and Random Forest (RF), are appealing for image classification, as they do not rely on data distribution assumptions and generally produce higher classification accuracies [14,15]. SVM classifiers have proven to be effective in tree species classification [16,17,18], along with RF classifiers [11,19].

As a subfield of machine learning, deep learning (DL), which attempts to model high-level abstractions in data using a hierarchal manner, has gained recent popularity in the remote sensing field for its ability to characterize complex patterns within imagery datasets. Similar to the function of the deep architecture of the human brain, deep learning algorithms formulate learning models that construct natural relationships between input and output data through deep architecture comprised of multiple layers of nonlinear transformation operations [20]. Contrary to the popular object-based approach to individual tree species classification, deep learning eliminates the hand-crafted feature extraction step, by examining the local spatial arrangement and structural patterns characterized by the low-level features [21]. Deep learning has demonstrated superior results over other commonly used classifiers for scene classification [22,23] as well as outperforming other methods in 3-D LiDAR tree species classification [24]. This study examines one of the latest neural networks for visual object recognition, Dense Convolutional Network (DenseNet), and its ability to classify dominant tree species within a highly complex urban environment using a data fusion approach with high spatial resolution multispectral imagery and LiDAR datasets. Recent studies have demonstrated DenseNet outperforms other deep learning architectures such as Inception, VGG and ResNet through achieving higher classification accuracies with fewer input parameters [25,26,27]. While deep learning has recently exhibited success for individual tree detection [6], crop classification [28] and hyperspectral image classification [29], DenseNet has not been examined for its utility for individual tree species classification, to our knowledge.

The goal here is to evaluate high spatial resolution imagery in combination with LiDAR data for tree species classification in a complex urban environment, demonstrated within a highly biodiverse city park, Forest Park, in St. Louis, Missouri, USA, which represents an urban forest containing typical tree species found in big cities. Furthermore, the tree arrangement of the park is similar to normal urban tree distribution, where trees can be found near walkways, roads, buildings (residential and commercial), green spaces and can exist individually or in clusters of same or varied species. Crown sizes vary greatly between species as well as growth stage, which makes it difficult to distinguish individual tree spectral and spatial characteristics from moderate spatial resolution imagery. Therefore, higher spatial resolution data is required to identify single tree crown spectral and structural parameters needed for individual urban tree species classification. Moreover, a pixel-based classification method cannot be used for species classification due to high variation of spectral response within a single canopy [30]. 

The objectives of this study are to: (1) propose a data fusion approach with DenseNet for tree species identification. To best of our knowledge, DenseNet is the first time employed for urban tree specifies classification in this paper; (2) analyze the impact of different combination of data source such as PAN band, VNIR, SWIR, and LiDAR on detailed tree species classification, and the contribution of different features types extracted from different sensors; (3) compare DenseNet performance to SVM and RF classifiers and (4) investigate the impacts of the limited number of training samples on classification accuracy for various classifiers.

## 2. Materials and Methods

### 2.1. Study Area

**Figure 1:**
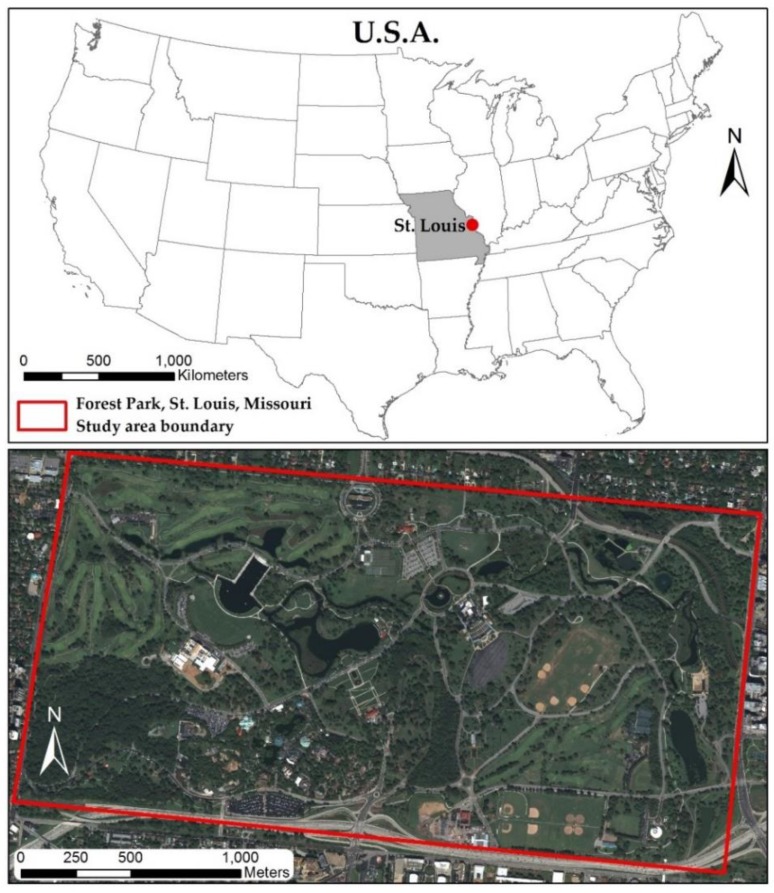
Study area located at Forest Park in St. Louis, MO, USA. The red border indicates the boundary of the park.

The study area is a 523 ha urban public park located in the western part of the city of St. Louis, MO, USA (Figure 1). Nestled amongst a city where 80% of the land has been developed for business, industry or residential uses, Forest Park serves as an important source of green space as well as an integrated ecosystem where humans and nature interact. Our study site, Forest Park, is home to more than 240 tree species interspersed among monuments, historic buildings, wildlife, waterways creating an ideal landscape for remotely sensed vegetation research [31]. According to a recent St. Louis Urban Tree Canopy Assessment, urban tree canopy coverage within the city of St. Louis is 18.2% (2929 ha) [32]. Within the study area, eight urban tree species (Table 1) were selected for this analysis based on the pervasiveness of that species (i.e., number of reference samples), as well as the ability to distinguish individual tree crowns on WV2, WV3 and LiDAR imagery. The selected trees, situated within the park boundary, are located along streets, near buildings and other high pedestrian areas, thereby representing the typical distribution of trees in an urban area. 

### 2.2. Datasets

Cloud-free WV2 VNIR and WV3 SWIR images acquired on 12 September 2012 and 21 August 2015, respectively, were used in this study. Image acquisition dates were selected based on both data availability and the vegetation growing season cycle in St. Louis. Cloud-free data over the study area was selected during leaf-on conditions and similar phenological stages. Although such a pair of images was not available within the same year, it is reasonably certain that the WV2 and WV3 images were acquired at similar seasonal vegetation growth phases. In St. Louis, mid-September belongs to late summer when trees reach maturity and develop a fully green canopy. WV2 and WV3 satellites collect VNIR data consisting of one panchromatic band (450–800 nm) with Ground Sampling Distance (GSD) of 0.5 m, and eight multispectral bands including coastal (400–450 nm), blue (450–510 nm), green (510–580 nm), yellow (585–625 nm), red (630–690 nm), red edge (705–745 nm), NIR1 (770–895 nm) and NIR2 (860–1040 nm). WV2 VNIR imagery has a spatial resolution of 1.84 m whereas WV3 VNIR imagery has a spatial resolution of 1.24 m. Eight additional shortwave infrared (SWIR) bands are offered through the WV3 satellite at 7.5 m spatial resolution. Although the WV3 sensor offers 16 multispectral bands, only SWIR data was available over the study area during leaf-on conditions. Both images were geometrically corrected and projected to WGS-84 UTM Zone 15N system. LiDAR data was obtained from the U.S. Geological Survey (USGS) through EarthExplorer and from the Missouri Spatial Data Information Service (MSDIS) in *LAS* format and processed in ArcGIS. LiDAR data for the study area was obtained on 22 December 2012 with average point spacing of 0.704 points per square meter and processed in ArcGIS at 1.5 m spatial resolution. WorldView 2/3 imagery is collected over the study area multiple times a year, eliminating the costly expense of tasking a satellite and the high spatial resolution (0.5 m pan-sharpened) is sufficient to distinguish individual tree canopies. Airborne LiDAR imagery is collected over the St. Louis Metropolitan area every 4–5 years. The collection strategies of these sensors allow for the repeatability of this methodology to update inventory datasets to better inform urban foresters, planners and managers. These datasets are outlined in Table 2.

### 2.3. Reference Data

Extensive mapping of tree species and their geographic locations throughout Forest Park has been conducted by the St. Louis City Department of Parks and Recreation and is updated annually. A detailed ground survey of trees located in Forest Park was conducted in September 2015 to validate locations provided by the Parks Department. An accompanying arborist provided tree species, tree height, tree condition, and potential fall risk. A total of 201 samples were surveyed at five locations within the park to independently verify the Parks Department tree species dataset. These data were aggregated into ArcGIS online using an iPad and Trimble R1 GNSS Receiver to tag the GPS location of each tree. All trees collected during the ground survey were verified against the Parks Department dataset and matched with 100 percent accuracy.

**Figure 2:**
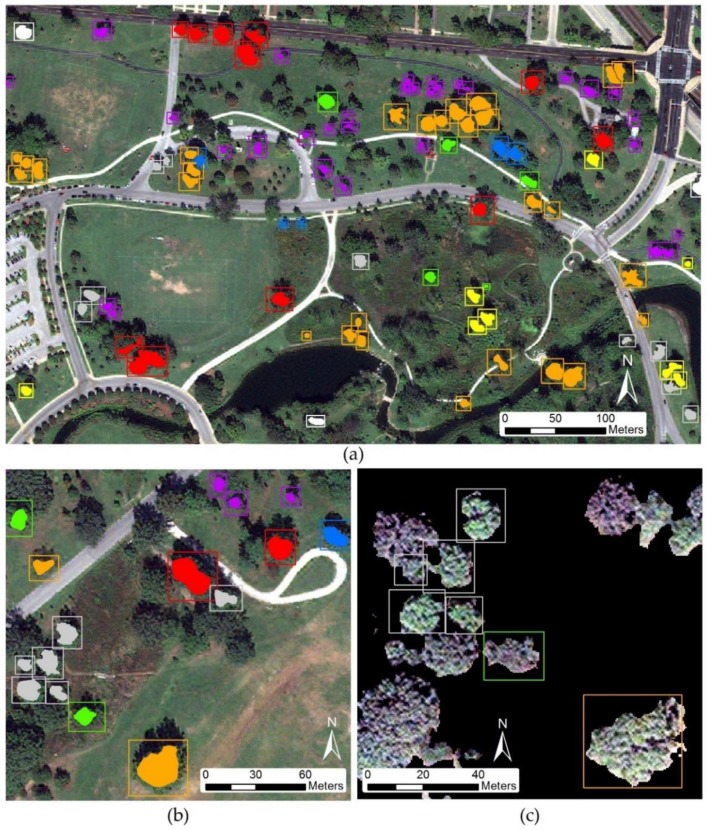
Manually delineated ground truth samples of dominant tree species within study area. (**a**) Small scale view of tree species reference sample subset. (**b**) Large scale view of reference samples for individual tree canopies. (**c**) Individual tree species samples after background removal.

A total of 1552 polygons were manually outlined in the Forest Park study area, of which a small subset is depicted in Figure 2a. Each polygon may consist of either an individual tree crown or a group of adjacent trees of the same species; if a group of trees of the same species cannot be visually separated on the image, one polygon may cover several crowns. Since WV2 and WV3 images were 3 years apart, all polygons were verified against the WV3 SWIR image to verify the continued presence of a tree. Additionally, individual tree occurrence within both datasets was verified against the Parks Department inventory records which are continuously maintained to document when a tree is cut down, dies, or replaced. As demonstrated in Figure 2b, rectangular polygons were created to include the entire boundary of the tree canopy to allow for the DenseNet algorithm to incorporate edge effects in its analysis. Given this workflow removes non-tree background prior to classification, this approach proved acceptable as it compares all classifiers against the same training sample conditions. After background removal, the only information remaining in each polygon, except for background pixels that eluded the threshold for removal, should equate to tree canopies. The background pixels were converted to null data and would not influence the SVM and RF classifiers, thus allowing us to utilize the rectangular polygon approach in the place of a polygon representing each individual tree canopy (Figure 2c). In the scenario where the rectangular polygon overlapped another canopy, it is acknowledged that this inclusion of extra information would affect all classifiers equally. 

### 2.4. Methods

**Figure 3:**
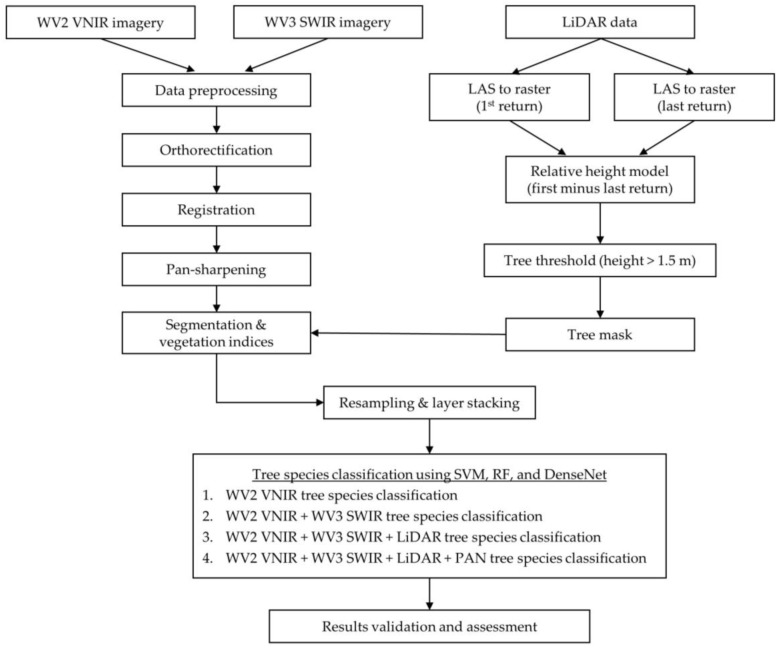
Classification workflow.

Figure 3 presents the flowchart of the urban tree species classification procedure using machine learning algorithms. The procedure consists of four steps: (1) data preprocessing; (2) tree crown area extraction; (3) shadow/background removal; and (4) tree species classification using machine learning algorithms SVM and RF and DenseNet. In order to evaluate the effect of data dimensionality on urban tree species identification, four classification schemes were tested: (1) classification based solely on the WV2 VNIR bands; (2) WV2 VNIR with WV3 SWIR bands; (3) WV2 VNIR, WV3 SWIR and LiDAR intensity image; and (4) WV2 VNIR, WV3 SWIR, LiDAR intensity and incorporation of the very high spatial resolution panchromatic (PAN) band from the WV2 dataset. An accuracy assessment was then performed for each classification scheme. 

#### 2.4.1. Data Pre-Processing

Each of the WV2 panchromatic/multispectral and WV3 SWIR Digital Number (DN) images was converted to Top of Atmosphere (TOA) radiance based on radiometric calibration parameters and standard correction formula using ENVI 5.4.1 software (Exelis Visual Information Solutions, Boulder, CO, USA) [33]. During radiometric calibration, raw digital numbers are rescaled to quantized calibrated pixel value in radiance (µW/ [cm^2^ · sr · nm]). Atmospheric correction was performed using ENVI’s Fast Line-of-Sight Atmospheric Analysis of Spectral Hypercubes (FLAASH) algorithm, which incorporates the MODTRAN (MODTRAN5v2r1) radiation transfer model [34]. A mid-latitude summer atmospheric model and an urban aerosol model were selected based on a seasonal-latitude surface temperature model and scene-appropriate standard MODTRAN aerosol model, respectively [35]. A scene visibility of 40 km, indicating clear weather conditions on image date, was chosen to calculate the aerosol amount and estimate a scene-average visibility using a dark pixel reflectance ratio method [36]. The resulting output was WV2 multispectral and WV3 SWIR surface reflectance images.

**Figure 4:**
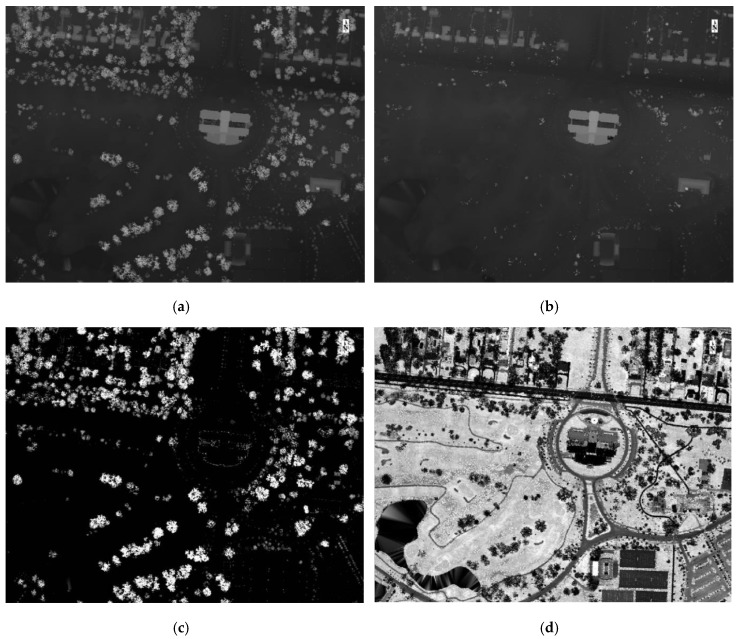
LiDAR data processing steps. (**a**) First return LAS data. (**b**) Last return LAS data. (**c**) Relative height model (last return subtracted from first return). (**d**) LiDAR intensity return image.

LiDAR data can be used to extract certain biophysical tree parameters essential for sustainable forestry management, such as, diameter at breast height (DBH), forest biomass, forest density, crown base height and tree height [37,38,39]. Ground truth tree height values have been compared to LiDAR derived tree height and were found to exhibit high *r*^2^ values, 0.85–0.95, thus establishing LiDAR as a valid technique to obtain tree height [40]. The height of trees can be determined by creating two files from the LiDAR dataset: a digital terrain model (DTM) and digital surface model (DSM). The DTM consists of the last returns of a LiDAR dataset and represents the bare earth. The DSM consists of all other returns and represents features on the surface of the earth. Subtracting DTM values from their corresponding DSM values is an accepted method for establishing the height of landscape features such as trees [41,42]. A similar approach was applied by converting LAS point cloud data into first return (Figure 4a) and last return (Figure 4b) raster images representing DSM and DTM raster images, respectively. By subtracting last return LiDAR raster from first return LiDAR raster, a relative height raster image depicting objects within the scene is produced (Figure 4c). Additionally, a LiDAR intensity return image (Figure 4d) was created to examine the usefulness of return intensity, a measure of object reflectivity, for distinguishing tree species. To extract tree-related information, a mask was created to exclude objects below 1.5 m, eliminating shrubs and flat or low-pitched roofs, and above 35 m, corresponding to the maximum height of native trees as well as excluding tall buildings and structures. This approach is effective in an urban area due to its ability to exclude buildings and houses, where most the remaining information relates to the relative height and intensity returns from woody vegetation. 

The WV2 and WV3 images were geometrically corrected using ENVI 5.4.1 software which employs a rational polynomial coefficients (RPC) based orthorectification technique by generating ground control points (GCPs) from an orthorectified reference image. Digital aerial ortho photos from the National Agriculture Imagery Program (NAIP) acquired on 18 June 2012 at 1m ground sample distance (GSD) were used as our reference image along with a U.S. Geological Survey (USGS) 1m digital elevation model (DEM) as the reference DEM. A total of 44 GCPs were created across the study area with a root mean square error (RMSE) of less than one pixel (2 m). The resulting orthorectified WV2 and WV3 images and LiDAR intensity images were then registered, via ENVI software’s image to image registration, to the WV2 panchromatic (PAN) image to geometrically align images and eliminate displacement caused by differences in image acquisition time and satellite observation angle to ensure corresponding pixels represent the same tree crowns [43]. Tie points for each image were manually created at prominent land features (e.g., road/path intersections, landmarks, etc.) and evenly distributed across the study area. At least 100 tie points were created for each image pairing achieving an RMSE of less than 0.5 m for each registration.

The co-registered WV2 multispectral surface reflectance image was fused with the WV2 panchromatic image using ENVI software’s Gramm-Schmidt Pan Sharpening tool with cubic convolution resampling method to create a high spatial resolution 0.5 m WV2 multispectral reflectance image [44]. Pan-sharpening algorithms are used to sharpen multispectral data using high spatial resolution panchromatic data. When sharpening WV2 imagery, the Gramm-Schmidt method has demonstrated better results at retaining spectral quality over other pan-sharpening methods [45]. The Gramm-Schmidt pan-sharpening method has been utilized in various tree species classification studies [4,5,46] due to its ability to improve delineation of individual tree crowns. WV2 orthoimages were then segmented into patches using ENVI software’s segment feature extraction tool to extract at total of 118 feature variables, including 64 statistical spectral features (i.e., maximum, minimum, mean, and standard deviation spectra of pixels forming a region in a specific band), 40 textural features, and 14 shape features were extracted (Table 3). For canopy segmentation, the WV2 multispectral orthoimage was used as the input dataset and the intensity scale parameter was set to 0 with the full lambda merge algorithm set to 87 and a texture kernel size of 3 [47]. In addition, 13 commonly used vegetation indices were generated to supplement the feature information (Table 4). Finally, the orthorectified WV2/WV3 imagery, LiDAR intensity, texture and vegetation indices were stacked and resampled to a 0.5 m spatial resolution using the cubic convolution resampling method with ENVI 5.4.1 software package. Resampling of the SWIR data from 7.5 to 0.5 m was necessary in order analyze all data at a uniform spatial resolution using a data fusion approach, a method which has been employed in similar data fusion studies [28]. The statistical spectral features along with the vegetation indices were examined for their potential to distinguish spectral variation between tree species. Texture and shape features were considered because crowns of different tree species have varied crown structures, shapes, and canopy densities.

#### 2.4.2. Background Removal

A stratified threshold approach was used to remove background and shadows that were still represented in the image following the LiDAR-derived tree mask. First, a bimodal histogram threshold method [68] was utilized to segment tree crowns from remaining background (e.g., road, pathway, grass, etc). Non-vegetation background was removed using a threshold statement where the two bands compared corresponded to the peaks and valleys representing the standard vegetation spectral curve. Next, tree canopy objects were delineated from the other vegetation background such as grass using thresholds determined from a stepwise approximation method [4]. Due to the active nature of LiDAR collection, shadows are not distinguished and were, thus, unable to be separated during the LiDAR relative height masking process. Shadow removal was important in this study as it can prove difficult to retrieve accurate spectral information from shadowed tree canopies. Shadows were removed using a bimodal histogram threshold method determined through comparison of the histogram of the NIR1 band image to separate shadowed and non-shadowed pixels. Pixels with NIR1 reflectance values higher than the threshold were retained as the non-shadowed pixels, while shadowed pixels were excluded from the image. Studies have shown that reflectance in shadowed regions is significantly less in the NIR band than sunlit areas [69]. 

#### 2.4.3. Classification Methods

Three different classifiers were applied on each data set, namely: (1) DenseNet [25]; (2) RF [70]; and (3) SVM [71]. The classifiers were chosen for to their ability to classify high-dimensional datasets. Our objective is to test the accuracy these three machine learning architectures for classifying tree species within a highly complex urban image. The effectiveness of each classifier will be examined under restricted training sample sizes.

Convolutional neural networks (CNNs) have become the dominant machine learning approach for object recognition and are becoming increasingly popular in the remote sensing field. Similar to the function of the human brain, CNNs are made up of neurons with learnable weights and biases. Each neuron receives several inputs, takes a weighted sum over them, passes it through an activation function and responds with an output [72]. The four key components of CNNs are convolution, activation, pooling, and fully connected layers. The main building block of CNNs is the convolutional layer, which utilizes a convolution filter, or kernel, to extract features of an image, beginning with low level and moving towards high level, or more complex, attributes. To account for non-linearity of the neurons, the result of the convolution function is passed through an activation function in the activation layer. Following the convolution operation, pooling is performed to reduce the dimensionality. Pooling layers down sample each feature map created from the convolution layer, reducing the input dimension, while retaining depth. This process decreases the number of parameters which cuts processing time and reduces overfitting. The last layer in the CNN is fully connected, meaning that neurons of preceding layers are connected to every neuron in subsequent layers. The input to the fully connect layer is a flattened after the final pooling layer is a 1-dimensional vector, ultimately created from a series of convolution and pooling layers.

**Figure 5:**
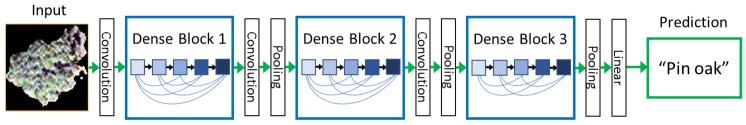
A deep DenseNet with three dense blocks. The layers between two adjacent blocks are referred to as transition layers and change feature-map sizes via convolution and pooling.

DenseNet, an adaptation of CNN, is a feed-forward artificial neural network designed to ensure maximum information flow between layers in the network. Differing from other CNNs, DenseNet connects all layers, to include matching feature map dimensions, directly to each other. Other CNNs may suffer from a drop out of input information as layers move deeper into the network. As a measure to preserve information flow between layers, each layer receives additional inputs from all preceding layers and passes on its own feature maps to all subsequent layers [25] (Figure 5). 

DenseNet combines features through concatenation rather than summation, like other CNNs. Where traditional CNNs have connections in a layer network, DenseNet has $\frac{N\left(N+1\right)}{2}$, in which each layer is connected to every other layer in a feed-forward manner. Other deep learning methods were not examined in this study as DenseNet has been widely demonstrated to outperform other CNNs in most applications [25]. The DenseNet architecture in this study utilized a 3-layer dense block with a growth rate of 4, depth of 40 and 100 epochs, without any dropout. 

To validate the effectiveness of DenseNet, its performance was compared with two popular machine learning classifiers: RF and SVM. The RF classifier is a non-parametric ensemble method that constructs a batch of individual decision trees (e.g., 500 in this study) where each decision tree outputs a class, which, if designated as the majority class, is assigned to the instance to be classified [70]. SVM is effective for solving non-linear, high dimensional space classifications [71]. SVM is effective at handling complex classifications, such as multispectral and hyperspectral, where spectral differentiation of target features may prove difficult. SVM is a supervised machine learning algorithm which can be used for classification and regression analysis. Using assigned training data, linear SVM creates a model that transforms the data into classes, based on a user-defined kernel function (e.g., RBF kernel in this study), then finds an optimal hyperplane that maximizes the margin distance between classes. Since it can find the optimal hyper-plane in high dimensional feature space, SVM is often used for classification of complex datasets.

## 3. Results

### 3.1. Classification Accuracy Using a Data Fusion Approach

To identify the optimal data fusion approach to use for the comparison of machine learning classifiers, the combination of WV2 Panchromatic and VNIR band, WV3 SWIR and LiDAR datasets were tested for classification accuracy. DenseNet was used to classify the eight dominant tree species using 8 pan-sharpened VNIR bands of the WorldView-2 image, then adding 8 SWIR bands of the WorldView-3 image, LiDAR return intensity image, and finally the panchromatic band from the WorldView-2 dataset. The fully combined dataset consisted of 18 bands. Table 5 shows the results for the DenseNet classification using a data fusion approach. 

The addition of each dataset improved overall classification demonstrating the ability of DenseNet to extract useful information from each dataset. Overall and average accuracies increased with each additional dataset starting at 75.9% and 71.2% and improving to 82.6% and 80.9%, respectively. The kappa coefficient, which is a statistical measure of inter-rater reliability, was also improved with each added combination, ranging from 0.71 when using only 8 VNIR bands to 0.80 when incorporating all 18 combined bands from three different sensors. The highest total overall accuracies were achieved using a fused combination of 18 bands derived from WorldView-2, WorldView-3 and LiDAR datasets.

Individual tree species varied in the classification accuracies depending on the combination of datasets demonstrating the unique spectral or textural characteristics of each species identified by different sensors. Green ash classification was increased significantly from 34.9% to 81.4% with the addition of the 8 SWIR WorldView-3 bands to the 8 VNIR WorldView-2 bands. However, the incorporation of LiDAR and panchromatic bands decreased accuracy to 62.8% and 60.5%, respectively, demonstrating its spectral separability in the SWIR region. Classification accuracy for sycamore was decreased with the addition of datasets to the initial 8 VNIR WorldView-2 bands from 85.9% to 71.8%, indicating its spectral separability in the VNIR region. Among all the species, bald cypress produced the highest classification of 97.6% with the incorporation of the LiDAR intensity image highlighting its unique structural characteristics exhibited from leaf-off LiDAR intensity returns. Three of the eight species, green ash, bald cypress and sycamore, did not achieve the highest classification accuracy with the addition of the panchromatic band to the other combined datasets. The decrease in accuracy with the incorporation of additional dataset highlight the potential for classification confusion resulting from redundancy of information with the panchromatic band and the pan-sharpened VNIR bands.

SVM and RF classifiers are frequently used for tree species classification [5,8,11] due to their capacity to deal with high-dimensional datasets. The SVM classifier utilized Radial Basis Function Kernel (RBF) as the kernel function with a three-fold cross grid search method to determine optimal classifier parameters. Validation results for the RF classifier set the optimal decision tree parameter at 500 trees. Individual tree species classification results from commonly used machine classifiers are presented in Table 6.

Similar to results achieved from the DenseNet classifier, overall accuracies, albeit much lower, increased with the addition of each dataset ranging from 48.2% to 51.8% and from 48.6% to 52% for SVM and RF classifiers, respectively. Likewise, kappa coefficients were lower for SVM and RF classifiers ranging from 0.39 to 0.44 for SVM and 0.38 to 0.42 for RF, which can be interpreted as fair to moderate per the aforementioned scale. Despite higher overall accuracies, RF produced lower kappa coefficients, potentially due to its inability to accurately classify cottonwood species. Pin oak and Austrian pine were among the highest individual species classification accuracies for both classifiers achieving 73% and 75.7% and 92.3% and 82.9% for SVM and RF, respectively. Overall accuracy was highest for the SVM classifier at 51.8% when using the full 18-band combination of VNIR/SWIR/LiDAR/Pan datasets while the RF classifier produced the highest accuracy of 53.1% with the exclusion of the panchromatic band. 

### 3.2. Classification Results Incorporating Vegetation Indices and Textural Information

Since the highest overall classification accuracy was achieved for two of the three classifiers—DenseNet (82.6%) and SVM (51.9%)—with the full combination of 18 bands (8 WV2 VNIR, 8 WV3 SWIR, LiDAR intensity return, WV2 panchromatic band), this dataset was chosen as the common dataset to compare the addition of the designated 13 VIs (Table 4) and 118 extracted statistical spectral features, textural features, and shape features (Table 3) for each classifier. Generally, the addition of VIs and textural features to the 18-band data fusion set increased the classification accuracy of individual species for the SVM and RF classifiers. Except for cottonwood, the incorporation of the 13 VIs increased SVM classification accuracy for individual species as well as the overall accuracy (60%), average accuracy (54.9%) and kappa coefficient (0.53). Adding the 118 features to the SVM classifier decreased overall accuracy (58.3%) and kappa coefficient (0.51).

For the RF classifier, the inclusion of the 13 VIs increased classification accuracy for five of the eight tree species while only slightly decreasing classification accuracy for Bald cypress (40.5%), Sugar maple (15.8%) and pin oak (65.4%) species. Conversely, the addition of the 118 features increased classification accuracy for the same individual species (Bald cypress—59.5%, Sugar maple—36.8%, Pink oak—94.2%) while simultaneously decreasing classification accuracy for Green ash (20.9%), Austrian pine (84.3%) and Sycamore (65.4%) tree species. Overall accuracy, average accuracy and the kappa coefficient improved with the incorporation of the 13 VIs and subsequently with the inclusion of the 118 statistical spectral features, textural features, and shape features.

Unlike the SVM and RF classifiers, DenseNet performance generally decreased with the addition of extra information to the combined 18-band dataset (VNIR/SWIR/LiDAR/Pan). Green ash was the only species to increase individual classification accuracy with the integration 13 VIs and 118 features (textural + statistical spectral + shape) to 65.1% and 74.4%, respectively. There was no change in classification accuracy for bald cypress (92.9%) when adding the 13 VIs but decreased to 78.6% with in inclusion of the 118 features for the DenseNet classifier. The incorporation of the 13 VIs improved classification accuracy for sycamore (89.7%) but decreased to 84.6% when adding the 118 extracted features, which is still an improvement over the 18-band combination (71.8%). Oppositely, Austrian pine decreased in classification accuracy (82.8%) with the assimilation of the 13 VIs but improved to its highest classification accuracy (90%) with the inclusion of the 118 features. Supplementation of the initial 18-band dataset decreased overall performance when using the DenseNet classifier, with overall accuracy, average accuracy, and kappa coefficient decreasing with the incorporation of each additional dataset.

**Figure 6:**
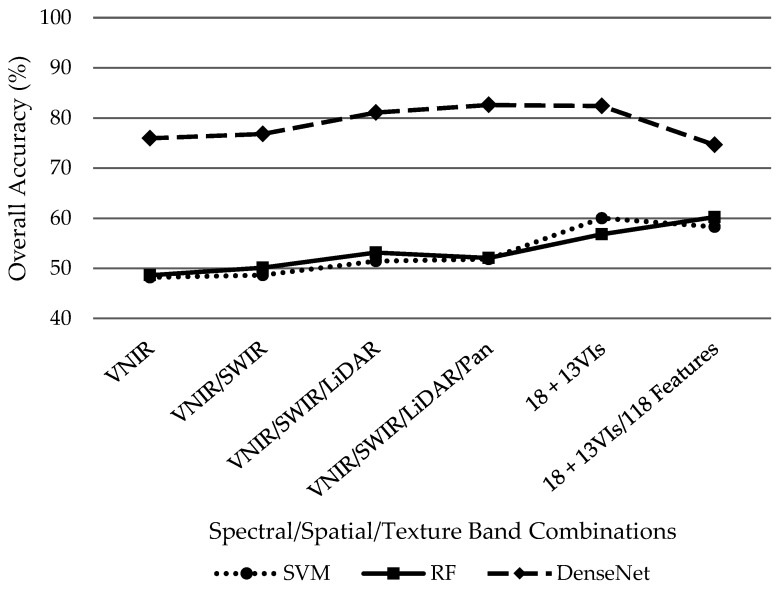
Overall accuracies for SVM, RF and DenseNet classifiers using various dataset combinations.

Overall, DenseNet outperformed SVM and RF classifiers regardless of the dataset combination as demonstrated in Figure 6. However, it is worth noting that the addition of supplementary datasets improved overall accuracy from the initial 18-band VNIR/SWIR/LiDAR/PAN dataset for the SVM and RF classifiers while the additional information hindered DenseNet performance by decreasing overall accuracy after the initial 18 bands. Using the initial 18 bands, SVM and RF classifiers demonstrated similar performance with overall accuracies of 51.8% and 52%, respectively. The SVM classified performed its best (60%) while only including the 13 VIs without the 118 features. Conversely, the RF classifier slightly outperformed SVMs highest accuracy with 60.2% with the incorporation of 13 VIs plus the 118 features despite underperforming the SVM classifier with only the initial 18 bands plus 13 VIs at 56.8%. Distinctively, DenseNet achieved its best results 82.6% while only incorporating the initial 18 VNIR/SWIR/LiDAR/PAN bands. Overall accuracy then decreased with the addition of 13 VIs then the 118 features to 82.4% and 74.6%, respectively. 

## 4. Discussion

### 4.1. Data Fusion for Urban Tree Species Classification

This study demonstrates the benefits of a data fusion approach to improve urban tree species classification. The optimal combination for classification of eight dominant trees species (reference samples >100) included eight multispectral WV2 VNIR bands, eight multispectral WV3 SWIR bands, one LiDAR intensity image band along with one panchromatic WorldView-2 band from the same data collection as the VNIR bands. These datasets were chosen due to their low cost, compared to hyperspectral datasets, and readily available nature. WV2 VNIR imagery has demonstrated the ability to successfully distinguish individual tree species [5,8] while WV3 imagery has demonstrated the potential for SWIR improving vegetation mapping across a heterogenous landscape [28]. LiDAR data can be used to extract individual canopies as well as estimate various structural parameters of trees, such as height, volume and biomass using both height and intensity returns [3,11]. Given the complex nature of tree canopies, object-based classification approaches are typically employed over pixel-based [8] to account for the varying illumination gradients that exist within an individual canopy and can affect spectral response.

Classification accuracies for individual species varied with incorporation of additional datasets and varied depending upon the classifier. Sycamore was the only species to produce the highest classification accuracy (85.9%) using only the eight WorldView-2 VNIR bands with the DenseNet classifier (Table 5). Using DenseNet, green ash achieved its highest classification accuracy (81.4%), a 46.5% increase, with the addition of 8 WV3 SWIR bands to the eight WV2 VNIR bands. Classification accuracy for green ash then decreased to 62.5% and 60.5% with the incorporation of LiDAR intensity data and the panchromatic band, respectfully. This is potentially explained by the effect of the emerald ash borer (*Agrilus planipennis*) infestation on the ash genus, potentially affecting the spectral response in the SWIR region which can be distinguished with the additional 8 SWIR bands offered by the WorldView-3 satellite. Generally, highest individual classification accuracy was obtained with the incorporation of the LiDAR intensity data or LiDAR with panchromatic band. Some species exhibited a decrease in accuracy with the inclusion of the panchromatic band, which is potentially due to the confusion added with the redundancy of information with the pan-sharpened VNIR bands. Overall, excluding the addition of the panchromatic band to the VNIR/SWIR/LiDAR datasets with the RF classifier, the overall accuracies increased with the combination of data from three different sensors. This demonstrates the potential for improved classification of complex heterogeneous landscapes using a data fusion approach. Each species responds uniquely to each sensor and the merger of information from varied datasets allows for a more comprehensive classification of diverse species.

### 4.2. Deep Learning vs. Other Commonly Used Classifiers

**Figure 7:**
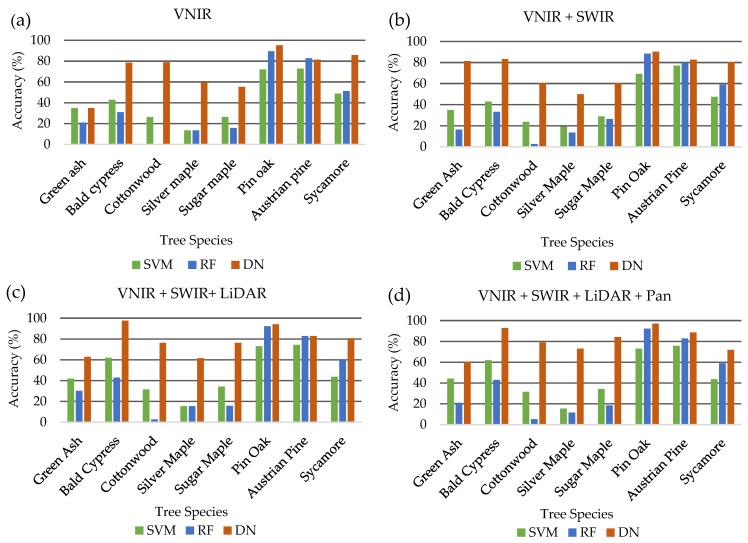
Overall accuracies for individual tree species using SVM, RF, and DenseNet (DN) classifiers using (**a**) eight VNIR WorldView-2 bands, (**b**) 8 VNIR WorldView-2 bands plus eight SWIR WorldView-3 bands, (**c**) eight VNIR WorldView-2 bands plus eight SWIR WorldView-3 bands plus LiDAR intensity return image and (**d**) eight VNIR WorldView-2 bands plus eight SWIR WorldView-3 bands plus LiDAR intensity return image plus WorldView-2 panchromatic band.

With the recent emergence and popularity of deep learning for object detection and image classification [6,22,24], this study sought to compare its effectiveness against other machine learning classifiers with proven success for tree species classification [5,8,11]. Figure 7 demonstrates the power of deep learning algorithms such as DenseNet to effectively classify individual trees species in a complex landscape. Apart from RF outperforming DenseNet for individual species classification of Austrian pine by 1.5% (82.9% > 81.4%), DenseNet outperformed all classifiers across all individual species classification accuracies. Furthermore, DenseNet produced higher overall and average accuracies along with higher kappa coefficients, indicating that the data collected are substantial representations of the presented variables according to Landis and Koch, 1977 [73]. The addition of VIs and 118 textural/statistical spectral/shape features increased performance of the SVM and RF classifiers, while decreasing the performance of DenseNet. This demonstrates the ability of deep learning classifiers such as DenseNet to extract additional information from input dataset without the added processing steps required for commonly used remote sensing image classifiers, such as SVM and RF, that are used with pre-extracted spectral and spatial (texture + shape) features. 

### 4.3. Contribution of Extracted Features on Classification Accuracy

To further explore the significance of feature type on classification accuracy, extracted features were divided into shape, statistical spectral and texture information categories. Each feature type category and group of 13 VIs (Table 4) was added separately to the 18-band fused dataset consisting of VNIR, SWIR, LiDAR and panchromatic datasets then classifications were performed using SVM, RF, and DenseNet classifiers with 70% training and 30% testing samples from the tree species sample dataset. The results are presented in Table 7 Apart from shape features with the SVM classifier (51%), the addition of each feature type increased overall classification accuracies as compared to exclusively using the original 18-band fused dataset. For RF classifier, texture features produced the highest overall classification accuracies of the three feature categories at 59.8%. Conversely, as demonstrated in Table 8 with the aggregate of feature types, the addition of separate feature types decreased overall classification accuracies for the DenseNet classifier. Furthermore, the addition of vegetation indices to the fused imagery dataset produced varied results across the classifiers. For SVM and DenseNet, the incorporation of VIs resulted in higher classification accuracies (60% and 82.4%, respectively) than any of the segmented feature types. Using the RF classifier, the supplementation of VIs produced a higher classification accuracy (56.8%) than shape and statistical spectral features, but was outperformed when only adding texture features to the fused dataset. 

Results for extracted feature types and VIs varied across all classifiers with different combinations performing better for certain classifiers than others. When adding another dataset category such as shape/statistical spectral/texture/VI to the 18-band fused imagery dataset, SVM and DenseNet generated highest overall classification accuracies (60% and 82.4%, respectively) with the incorporation of VIs. Under the same investigation, RF produced the highest overall classification while integrating texture feature with the 18-band fused dataset. Within each classifier, RF was the only classifier to achieve its highest overall classification accuracy of 60.2% when all datasets were combined. SVM produced its highest overall accuracy of 60% with the combination of VIs with the 18-band fused imagery dataset. Regardless of additional dataset category, DenseNet attained the highest overall classification accuracy of 82.6% for all classifiers and dataset combinations with the original 18-band fused imagery dataset. However, it should be mentioned that the separate inclusion of texture and VIs to the 18-band fused data only achieved mildly lower overall classification accuracies of 80.4% and 82.4%, respectively.

### 4.4. Impact of Training Samples on Classifier Performance

**Figure 8:**
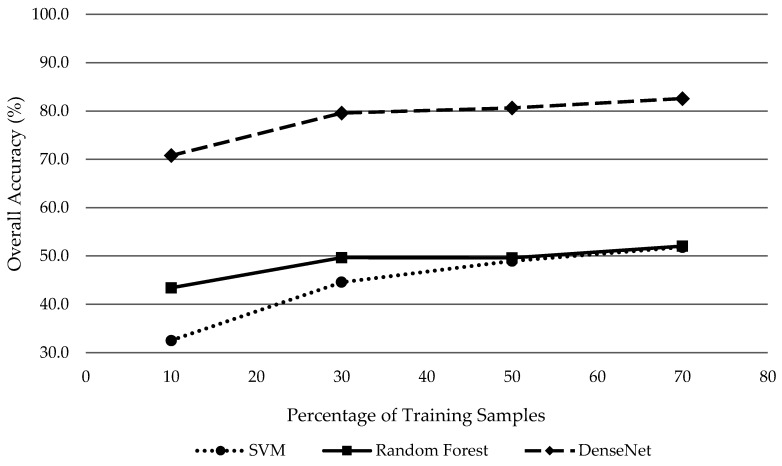
Overall accuracy for SVM, RF, and DenseNet classifiers using 10% training, 30% training, 50% training and 70% training from the total samples for each species using 18 bands.

Deep learning algorithms typically require a large sample set to build an effective model. Therefore, it was our hypothesis that while deep learning algorithms such as DenseNet outperform other machine learning classifiers given a robust sample dataset. In order to test the effect of the number training samples on classifier performance, each classifier’s ability to handle limited training samples was examined. The same training samples were used for each classifier to compare classifier performance against a matching set of variables. The results are displayed in Figure 8. A uniform percentage of the total samples for each tree species was chosen at twenty percent intervals starting at 10% and ending at 70%. The results are presented in Table 9.

Our results demonstrate that DenseNet outperformed other commonly used classifiers such as SVM and RF regardless of training sample numbers. SVM performed the poorest of all the classifiers when limited to 10% training samples with an overall accuracy of 32.5% (Table 9). The SVM classifier produced the lowest accuracy of all classifiers, regardless of training sample size, when attempting to classify eight classes of tree species with 18 total bands representing VNIR/SWIR/LiDAR/panchromatic datasets. SVM achieved similar, yet slightly lower (50% training = 49%, 70% training = 51.8%), overall accuracies as RF (50% training = 49.6%, 70% training = 52%) when training samples were 50% or higher of the total sample population. Both SVM and RF classifiers improved or produced similar classification accuracies with the increase of training sample size relative to total sample population. 

DenseNet produced significantly higher overall classification accuracies across all training sample size trials (Figure 8). Overall accuracies for DenseNet were on average 29.7% higher than the next closest classifier (RF). The increase of training sample size improved overall classification accuracy for the 18-band dataset, starting at 70.7% accuracy for 10% training samples of the class sample population and improving to 82.6% for a 70% training sample size (Table 9). In general, all classifiers improved with the increase of training sample size but DenseNet significantly outperformed SVM and RF classifiers across all training sample trials as shown in Figure 8. This demonstrates DenseNet is capable of extracting a variety of information from provided datasets and its robustness to number of samples used for training. Noticeably, increasing training samples improved DenseNet performance to achieve a more accurate classification of individual tree species. For this reason, only tree species with 100+ ground truth samples collected within our study site were utilized, otherwise there was not a sufficient test sample population of others species to accurately compare multiple classifiers. 

## 5. Conclusions

This study examined high spatial resolution imagery, i.e., WV2 VNIR and WV3 SWIR images, for analysis with an image-based classification method. At the study site, three classification schemes, including classification based on leaf-on WV2 VNIR images, both WV2 VNIR and WV3 SWIR images, and WV2/WV3 along with LiDAR derived tree extraction methods were conducted to examine the effects of high spatial resolution imagery and data fusion approaches on urban tree species classification. Two common machine learning algorithms, SVM and RF, were compared against the latest deep learning algorithm, i.e., DenseNet, to examine their ability to classify dominant individual tree species in a complex urban environment. Our results demonstrated that a data fusion approach, with the incorporation of VNIR, SWIR and LiDAR datasets improves overall accuracy of individual tree species classification across all classifiers employed in this study. 

We determined that DenseNet significantly outperformed popular machine learning classifiers, SVM and RF. The inclusion of additional variables (i.e., statistical spectral, textural, and shape features) hindered the overall accuracy of the DenseNet classifier while improving accuracy for RF and SVM for individual tree species classification. This indicates the strength of deep learning to analyze similar statistical spectral, textural and shape information within the hidden layers and without the need for engineering hand-crafted features. 

The contribution of each feature type on classification accuracy was investigated by separately adding shape, statistical spectral, texture, and VIs to the 18-band fused imagery baseline dataset. Among the individual input features, VIs added to the 18-band fused baseline dataset produced the highest overall classification accuracies with DenseNet (82.4%), which was followed by texture features (80.43%), and shape features (78.06%). Regardless of additional feature dataset category, DenseNet consistently attained the highest overall classification accuracy of 82.6% compared to SVM and RF. However, it should be mentioned that the separate inclusion of texture and VIs to the 18-band fused data achieved only mildly lower overall classification accuracies of 80.4% and 82.4%, respectively.

Moreover, limiting the amount of training samples, which counters deep learning’s position as the preferred classifier for large datasets with abundant training samples, DenseNet is still the superior classifier compared to SVM and RF for individual tree species classification. Regardless of the number of training samples, DenseNet outperformed with overall accuracies 29.7% higher on average than the next closest classifier (RF). This study demonstrates the potential of deep learning as a powerful classifier for complex landscapes such as urban tree species classification. However, to further explore its utility and robustness, deep learning algorithms should be tested at other study areas and across a variety of tree species and available datasets. 

## Figures and Tables

**Table 1:** List of tree species and number of training samples collected within the study area.

Tree Species (Common Name)	Scientific Name	Genus	Family	Class	Training Samples
Green ash	*Fraxinus pennsylvanica*	*Fraxinus*	Oleaceae	Deciduous	348
Bald cypress	*Taxodium distichum*	*Taxodium*	Cupressaceae	Deciduous	261
Cottonwood	*Populus deltoides*	*Populus*	Salicaceae	Deciduous	233
Silver maple	*Acer saccharinum*	*Acer*	Sapindaceae	Deciduous	172
Sugar maple	*Acer saccharum*	*Acer*	Sapindaceae	Deciduous	145
Pin oak	*Quercus palustris*	*Quercus*	Fagaceae	Deciduous	141
Austrian pine	*Pinus nigra*	*Pinus*	Pinaceae	Coniferous evergreen	126
Sycamore	*Platanus occidentalis*	*Platanus*	Platanaceae	Deciduous	126
				Total	1552

**Table 2:** Remotely sensed datasets.

WorldView-2	22 September 2012	Panchromatic: 450–800	Pan: 0.5 m VNIR: 2.0 m	11 bits per pixel
		Coastal: 400–450		
		Blue: 450–510		
		Green: 510–580		
		Yellow: 585–625		
		Red: 630–690		
		Red Edge: 705–745		
		NIR1: 770–895		
		NIR2: 860–1040		
WorldView-3	21 August 2015	SWIR-1: 1195–1225	SWIR: 7.5 m	14 bits per pixel
		SWIR-2: 1550–1590		
		SWIR-3: 1640–1680		
		SWIR-4: 1710–1750		
		SWIR-5: 2145–2185		
		SWIR-6: 2185–2225		
		SWIR-7: 2235–2285		
		SWIR-8: 2295–2365		
LiDAR	22 December 2012		1.5 m	
NAIP	18 June 2012	Blue: 400–580	VNIR: 1 m	11 bits per pixel
		Green: 500–650		
		Red: 590–675		
		NIR: 675–850		

**Table 3:** Feature variables (64 statistical spectral features, 40 textural features, 14 shape features).

Feature Name	Description	Datasets Utilized	Total Bands
SpecMean	Spectral mean value of pixels forming region in band *n*	VNIR/SWIR	16
SpecStd	Spectral standard deviation value of pixels forming region in band *n*		16
SpecMin	Spectral minimum value of pixels forming region in band *n*		16
SpecMax	Spectral maximum value of pixels forming region in band *n*		16
TextRange	Average data range of pixels comprising region inside kernel	VNIR/LiDAR/Pan	10
TextMean	Average value of pixels comprising region inside kernel		10
TextVar	Average variance of pixels comprising region inside kernel		10
TextEntro	Average entropy value of pixels comprising region inside kernel		10
Area	Total area of polygon, minus area of holes	Extracted Image	1
Length	Combined length of all boundaries of polygon, including boundaries of holes		1
Compactness	Indicates compactness of polygon [= √(4 * Area/π/outer contour length)]		1
Convexity	Measures convexity of polygon [= length of convex hall/length]		1
Solidity	Compares area of polygon to area of a convex hull surrounding polygon [= Area/area of convex hull]		1
Roundness	Compares area of polygon to square of maximum diameter of polygon [= 4 * Area/(π∗ Major_Length^2^)]		1
Form_Factor	Compares area of polygon to square of total perimeter [= 4 * Area/(π∗ total perimeter^2^)]		1
Elongation	Ratio of major axis of polygon to minor axis of polygon [= Major_Length/Minor_Length]		1
Rectangular_Fit	Compares area of polygon to area of oriented bounding box enclosing polygon [= Area/(Major_Length * Minor_Length)]		1
Main_Direction	Angle subtended by major axis of polygon and *x*-axis in degrees		1
Major_Length	Length of major axis of an oriented bounding box enclosing polygon		1
Minor_Length	Length of minor axis of an oriented bounding box enclosing polygon		1
Number_of_Holes	Number of holes in polygon in an integer value		1
Hole_Area	Ratio of total area of polygon to area of outer contour of polygon [= Area/outer contour area]		1
		Total	118

**Table 4:** Vegetation indices utilized for tree species classification.

Vegetation Index Name	Abbreviation	Formula	References
Atmospherically Resistant Vegetation Index	ARVI	$\frac{\mathrm{NIR}1-\mathrm{RED}-y\left(\mathrm{RED}-\mathrm{BLUE}\right)}{\mathrm{NIR}1+\mathrm{RED}-y\left(\mathrm{RED}-\mathrm{BLUE}\right)}$	[48]
Canopy Chlorophyll Content Index	CCCI	$\frac{\frac{\mathrm{NIR}1-\mathrm{Red}\;\mathrm{Edge}}{\mathrm{NIR}1+\mathrm{Red}\;\mathrm{Edge}}}{\frac{\mathrm{NIR}1-\mathrm{RED}}{\mathrm{NIR}1+\mathrm{RED}}}$	[49,50]
Green Normalized Difference Vegetation Index	GNDVI	$\frac{\mathrm{NIR}1-\mathrm{GREEN}}{\mathrm{NIR}1+\mathrm{GREEN}}$	[51,52,53]
Normalized Difference Red Edge Index	NDRE	$\frac{\mathrm{NIR}1-\mathrm{Red}\;\mathrm{Edge}}{\mathrm{NIR}1+\mathrm{Red}\;\mathrm{Edge}}$	[10,54]
Normalized Difference Red Edge Index—NIR2	NDRE2	$\frac{\mathrm{NIR}2-\mathrm{Red}\;\mathrm{Edge}}{\mathrm{NIR}2+\mathrm{Red}\;\mathrm{Edge}}$	[55]
Normalized Difference Vegetation Index	NDVI	$\frac{\mathrm{NIR}1-\mathrm{RED}}{\mathrm{NIR}1+\mathrm{RED}}$	[56,57,58]
Normalized Difference Vegetation Index—Green/Red Ratio	NDVI-GR	$\frac{\mathrm{NIR}1-\left(\mathrm{GREEN}+\mathrm{RED}\right)}{\mathrm{NIR}1+\left(\mathrm{GREEN}+\mathrm{RED}\right)}$	[59]
Normalized Difference Vegetation Index—Yellow	NDVI-Y	$\frac{\mathrm{NIR}1-\mathrm{YELLOW}}{\mathrm{NIR}1+\mathrm{YELLOW}}$	[60]
Normalized Difference Vegetation Index—NIR2	NDVI2	$\frac{\mathrm{NIR}2-\mathrm{RED}}{\mathrm{NIR}2+\mathrm{RED}}$	[61,62]
Normalized Difference Water Index	NDWI	$\frac{\mathrm{NIR}1-\mathrm{NIR}2}{\mathrm{NIR}1+\mathrm{NIR}2}$	[63,64]
Plant Senescence Reflectance Index	PSRI	$\frac{\mathrm{RED}-\mathrm{BLUE}}{\mathrm{Red}\;\mathrm{Edge}}$	[65]
Soil Adjusted Vegetation Index	SAVI	$\frac{\mathrm{NIR}1-\mathrm{RED}}{\mathrm{NIR}1+\mathrm{RED}+L}\times \left(1+L\right)$	[51,66]
Visible Atmospherically Resistant Indices—Red Edge	VARI-Red Edge	$\frac{\mathrm{Red}\;\mathrm{Edge}-\mathrm{RED}}{\mathrm{Red}\;\mathrm{Edge}+\mathrm{RED}}$	[67]

**Table 5:** Classification accuracies for eight dominant tree species using fused datasets and DenseNet classification using 30% training and 70% testing of each species total reference samples. All values, except for kappa coefficient, are percentages.

Tree Species	VNIR	VNIR+SWIR	VNIR+SWIR+LiDAR	VNIR+SWIR+LiDAR+PAN
Green Ash	34.88	81.40	62.79	60.47
Bald Cypress	78.57	83.33	97.62	92.86
Cottonwood	78.95	60.53	76.32	78.95
Silver Maple	59.62	50.00	61.54	73.08
Sugar Maple	55.26	60.53	76.32	84.21
Pin Oak	95.19	90.38	94.23	97.12
Austrian Pine	81.43	82.86	82.86	88.57
Sycamore	85.90	80.77	80.77	71.79
Overall Accuracy	75.91	76.77	81.08	82.58
Kappa Coefficient	0.72	0.73	0.78	0.80
Average Accuracy	71.22	73.72	79.05	80.88

**Table 6:** Classification accuracies for 8 dominant tree species using fused datasets with SVM and RF classifiers using 30% training and 70% testing of each species total reference samples. All values, except for the kappa coefficient, are percentages.

	VNIR	VNIR+SWIR	VNIR+SWIR+LiDAR	VNIR+SWIR+LiDAR+PAN
SVM Classification Accuracy				
Green Ash	34.88	34.88	41.86	44.19
Bald Cypress	42.86	42.86	61.90	61.90
Cottonwood	26.32	23.68	31.58	31.58
Silver Maple	13.46	19.23	15.38	15.38
Sugar Maple	26.32	28.95	34.21	34.21
Pin Oak	72.12	69.23	73.08	73.08
Austrian Pine	72.86	77.14	74.29	75.71
Sycamore	48.72	47.44	43.59	43.59
Overall Accuracy	48.17	48.60	51.40	51.83
Kappa Coefficient	0.39	0.40	0.43	0.44
Average Accuracy	42.19	42.93	46.99	47.46
RF Classification Accuracy				
Green Ash	20.93	16.28	30.23	20.93
Bald Cypress	30.95	33.33	42.86	42.86
Cottonwood	0.00	2.63	2.63	5.26
Silver Maple	13.46	13.46	15.38	11.54
Sugar Maple	15.79	26.32	15.79	18.42
Pin Oak	89.42	88.46	92.31	92.31
Austrian Pine	82.86	80.00	82.86	82.86
Sycamore	51.28	58.97	60.26	58.97
Overall Accuracy	48.60	50.11	53.12	52.04
Kappa Coefficient	0.38	0.40	0.43	0.42
Average Accuracy	38.09	39.93	42.79	41.64

**Table 7:** Classification accuracies for eight dominant tree species incorporating 13 VIs and 118 extracted features for SVM, RF, and DenseNet classifiers in addition to 18-band data fusion set (Base).

	SVM			RF			DenseNet		
Species	Base	VIs	VIs/Features	Base	VIs	VIs/Features	Base	VIs	VIs/Features
Green ash	44.19	51.16	51.16	20.93	30.23	20.93 **	60.47	65.12	74.42
Bald cypress	61.90	69.05	73.81	42.86	40.48 *	59.52	92.86	92.86	78.57 **
Cottonwood	31.58	26.32 *	31.58	5.26	7.89	15.79	78.95	68.42 *	55.26 **
Silver maple	15.38	23.08	36.54	11.54	21.15	34.62	73.08	67.31 *	57.69 **
Sugar maple	34.21	47.37	52.63	18.42	15.79 *	36.84	84.21	76.32 *	47.37 **
Pin oak	73.08	78.85	75.96 **	92.31	91.35 *	94.23	97.12	94.23 *	80.77 **
Austrian pine	75.71	77.14	65.71 **	82.86	88.57	84.29 **	88.57	82.86 *	90.00
Sycamore	43.59	66.67	53.85 **	58.97	73.08	65.38 **	71.79	89.74	84.62 **
Overall Accuracy	51.83	60.00	58.28 **	52.04	56.77	60.22	82.58	82.37 *	74.62 **
Kappa Coefficient	0.44	0.53	0.51 **	0.42	0.48	0.52	0.80	0.79 *	0.70 **
Average Accuracy	47.46	54.95	55.16	41.64	46.07	51.45	80.88	79.61 *	71.09 **

* Addition of VIs decreased classification accuracy from previous dataset; ** Addition of textural features/VIs decreased classification accuracy from previous dataset.

**Table 8:** Overall accuracies for SVM, RF, and DenseNet classifiers using fused imagery dataset combined with different segmented feature types or vegetation indices.

Datasets	SVM	RF	DenseNet
VNIR/SWIR/LiDAR/Pan (V/S/L/P)	51.83	52.04	82.58 *
V/S/L/P + Shape Features	50.97	56.13	78.06
V/S/L/P + Statistical Spectral Features	53.12	55.91	74.19
V/S/L/P + Texture Features	53.98	59.78	80.43
V/S/L/P + Vegetation Indices	60.00 *	56.77	82.37
V/S/L/P + All Features + VIs	58.28	60.22 *	74.62

* Highest classification accuracy of all data combinations per classifier.

**Table 9:** Overall accuracy for SVM, RF and DenseNet classifiers for eight dominant tree species using varying percentages of the total samples for each individual class.

	Overall Accuracy (%)		
Reference Sample %	SVM	RF	DenseNet
10	32.52	43.41	70.77
30	44.57	49.63	79.56
50	48.97	49.61	80.62
70	51.83	52.04	82.58
